# Supplementary material for: Effect of zinc deficiency on chronic kidney disease progression and effect modification by hypoalbuminemia
Source: PLoS One. 2021 May 11;16(5):e0251554. doi: 10.1371/journal.pone.0251554 (PMC8112700; doi:10.1371/journal.pone.0251554)
Supplement: S1 Table — (PDF) [file pone.0251554.s001.pdf]

S1 Table. Characteristics of patients after propensity score matching.

	All	Low-Zn group	High-Zn group	*p*
n	174	87	87	
**Demographic characteristic**				
Age (years)	69.6±13.8	69.2±14.6	70.0±13.0	0.71
Male (%)	96 (55.2)	50 (57.5)	46 (52.9)	0.65
BMI (kg/m^2)	22.7±4.2	22.2±3.3	23.2±4.8	0.09
**Comorbidity**				
Hypertension (%)	145 (83.3)	70 (80.5)	75 (86.2)	0.42
DM (%)	74 (42.5)	33 (37.9)	41 (47.1)	0.28
Dyslipidemia (%)	74 (42.5)	36 (41.4)	38 (43.7)	0.88
Cardiovascular disease (%)	69 (39.7)	34 (39.1)	35 (40.2)	1.0
Nephrotic syndrome (%)	23 (13.2)	13 (14.9)	10 (11.5)	0.66
Chronic liver disease (%)	19 (10.9)	9 (10.3)	10 (11.5)	1.0
Bowel disease (%)	1 (0.6)	0 (0.0)	1 (1.1)	1.0
Cancer (%)	40 (23.0)	17 (19.5)	23 (26.4)	0.37
**Medication**				
ARBs or ACE inhibitors (%)	77 (44.3)	36 (41.4)	41 (47.1)	0.54
Diuretics (%)	116 (66.7)	61 (70.1)	55 (63.2)	0.42
Zinc-containing drugs (baseline) (%)	6 (3.4)	1 (1.1)	5 (5.7)	0.21
Zinc-containing drugs (whole period) (%)	64 (36.8)	41 (47.1)	23 (26.4)	0.007
**Laboratory measurement**				
Zn (µg/dl)	61.0±12.8	51.0±6.6	71.0±9.0	<0.001
Creatinine (mg/dl)	2.45 (1.50, 4.42)	2.46 (1.51, 4.51)	2.37 (1.50, 4.22)	0.62

eGFR (ml/min/1.73m^2)	18.7 (10.2, 30.7)	18.5 (9.6, 31.3)	18.8 (11.7, 30.3)	0.71
CRP (mg/dl)	0.20 (0.09, 0.82)	0.20 (0.08, 0.99)	0.22 (0.10, 0.70)	0.90
Albumin (g/dl)	3.2±0.6	3.2±0.7	3.2±0.6	0.60
Hemoglobin (g/dl)	9.6±1.7	9.7±1.6	9.6±1.8	0.73
Dipstick proteinuria (≥1+) (%)	127 (73.0)	64 (73.6)	63 (72.4)	1.0

Continuous variables are shown as the mean ± SD or median (interquartile range). Categorical variables are shown as n (%). Abbreviations: Zn, serum zinc; BMI, body mass index; DM, diabetes mellitus; ARBs, angiotensin II receptor blockers; ACE, angiotensin-converting enzyme; eGFR, estimated glomerular filtration rate; CRP, C-reactive protein.
