# Supplementary material for: Effect of zinc deficiency on chronic kidney disease progression and effect modification by hypoalbuminemia
Source: PLoS One. 2021 May 11;16(5):e0251554. doi: 10.1371/journal.pone.0251554 (PMC8112700; doi:10.1371/journal.pone.0251554)
Supplement: S2 Table — (PDF) [file pone.0251554.s002.pdf]

S2 Table. Incidence of outcomes in patients grouped by Zn levels after propensity score matching.

	All	Low-Zn group	High-Zn group	*p*
n	174	87	87	
**Outcomes, n (%)**				
Primary outcome	52 (29.9)	33 (37.9)	19 (21.8)	0.031
ESKD	44 (25.3)	29 (33.3)	15 (17.2)	0.023
Hemodialysis	38 (21.8)	27 (31.0)	11 (12.6)	0.005
Peritoneal dialysis	6 (3.4)	2 (2.3)	4 (4.6)	0.68
Death	8 (4.6)	4 (4.6)	4 (4.6)	1.0
**Observational period (days)**	365 (93, 365)	323 (89, 365)	365 (103, 365)	0.28

Continuous variables are shown as median (interquartile range). Categorical variables are shown as n (%). Abbreviations: Zn, serum zinc; ESKD, end-stage kidney disease.
