# Supplementary material for: Effect of zinc deficiency on chronic kidney disease progression and effect modification by hypoalbuminemia
Source: PLoS One. 2021 May 11;16(5):e0251554. doi: 10.1371/journal.pone.0251554 (PMC8112700; doi:10.1371/journal.pone.0251554)
Supplement: S3 Table — (PDF) [file pone.0251554.s003.pdf]

S3 Table. Interaction between Zn levels and baseline characteristics.

Interaction terms	*p*
Zn × Age	0.30
Zn × Male	0.37
Zn × BMI	0.053
Zn × DM	0.036
Zn × Cardiovascular disease	0.55
Zn × ARBs or ACE inhibitors	0.25
Zn × Diuretics	0.49
Zn × ln(eGFR)	0.054
Zn × ln(CRP)	0.69
Zn × Albumin	0.026
Zn × Hemoglobin	0.69
Zn × Dipstick proteinuria	0.75

Each interaction term was evaluated using a multivariate Cox proportional hazards model adjusted for baseline characteristics. Each *p* value shows the result of each analysis. Abbreviations: Zn, serum zinc; BMI, body mass index; DM, diabetes mellitus; ARBs, angiotensin II receptor blockers; ACE, angiotensin-converting enzyme; eGFR, estimated glomerular filtration rate; CRP, C-reactive protein.
