# Supplementary material for: Effect of zinc deficiency on chronic kidney disease progression and effect modification by hypoalbuminemia
Source: PLoS One. 2021 May 11;16(5):e0251554. doi: 10.1371/journal.pone.0251554 (PMC8112700; doi:10.1371/journal.pone.0251554)
Supplement: S4 Table — (PDF) [file pone.0251554.s004.pdf]

S4 Table. Association of zinc-containing drugs with the primary outcome in subgroups according to Zn levels.

	Adjusted HR (95% CI)	*p*
Patients with low Zn levels	0.38 (0.21, 0.69)	0.001
Patients with high Zn levels	0.47 (0.17, 1.33)	0.16

Adjusted HRs of patients with zinc-containing drugs relative to patients without zinc-containing drugs are shown. HRs were adjusted for baseline characteristics, such as age, male sex, BMI, DM, cardiovascular disease, ln(eGFR), ln(CRP), serum albumin levels, hemoglobin levels, dipstick proteinuria, ARBs or ACE inhibitors, and diuretics.
