# Supplementary figures and images for: Effect of zinc deficiency on chronic kidney disease progression and effect modification by hypoalbuminemia
Source: PLoS One. 2021 May 11;16(5):e0251554. doi: 10.1371/journal.pone.0251554 (PMC8112700; doi:10.1371/journal.pone.0251554)

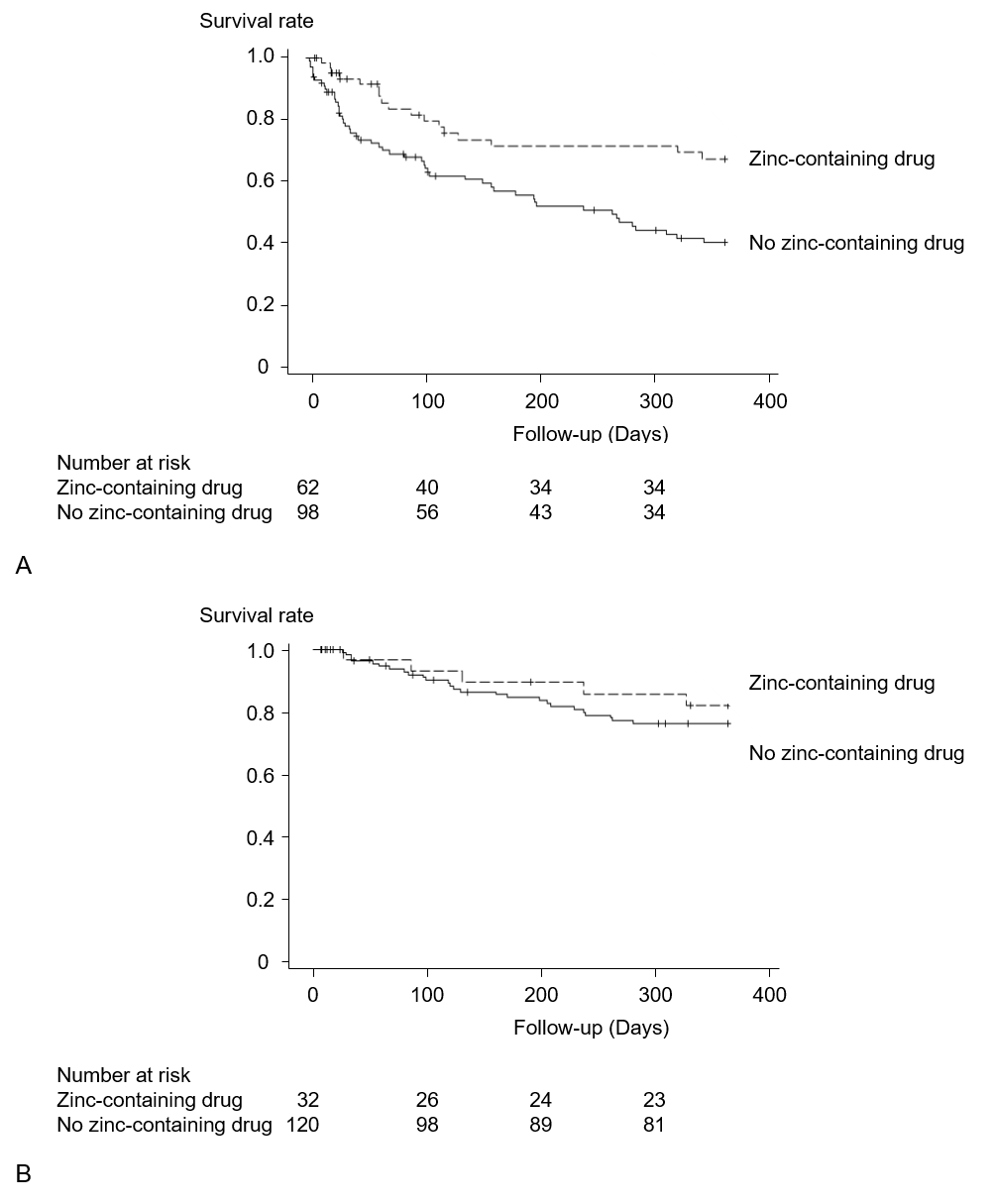

Supplement: S1 Fig — Kaplan-Meier curves for the primary outcome over 1 year. (A) Patients with low Zn levels (Zn level <60 μg/dl). (B) Patients with high Zn levels (Zn level ≥60 μg/dl). Survival rates were compared between the two groups (with and without zinc-containing drugs) by the log-rank test. (TIF) [file pone.0251554.s005.tif]
