# Supplementary material for: Effect of zinc deficiency on chronic kidney disease progression and effect modification by hypoalbuminemia
Source: PLoS One. 2021 May 11;16(5):e0251554. doi: 10.1371/journal.pone.0251554 (PMC8112700; doi:10.1371/journal.pone.0251554)
Supplement: Attachment — Submitted filename: comment to PONE-D-21-03589.docx [file pone.0251554.s006.docx]

Tokuyama et al. reported a retrospective cohort study that clarified zinc deficiency as a risk of CKD progression in patients with CKD, in which authors used cox proportional hazards models and propensity score-matched analysis. In addition, authors showed the association between serum zinc levels and albumin levels, and concluded hypoalbuminemia affected the relationship between zinc and outcomes. This study and statistical analysis were well designed, and clearly showed the effect of zinc deficiency on CKD progression.

Major comment

(1) In figure 6, authors presented the effect of zinc deficiency on outcome in low-albumin patients and high-albumin patients in which concluded no association between zinc deficiency and outcome in high albumin patients. However, it is not clear why there is the difference of zinc-associated outcome between both groups. Authors should describe the possible mechanisms or hypothesis that zinc deficiency affect the outcome in low-albumin groups.
(2) A title is “Effect of zinc deficiency on chronic kidney disease progression”. If there is no association of zinc deficiency and outcome in CKD patients with high albumin group, a title may mention the effect of hypoalbuminemia on the relationship between zinc deficiency and outcome.
